# Efficacy and safety of targeting VEGFR drugs in treatment for advanced or metastatic gastric cancer: a systemic review and meta-analysis

**DOI:** 10.18632/oncotarget.23429

**Published:** 2017-12-19

**Authors:** Duanrui Liu, Xiaoli Ma, Dongjie Xiao, Yanfei Jia, Yunshan Wang

**Affiliations:** ^1^ Central Laboratory, Jinan Central Hospital Affiliated to Shandong University, Jinan 250013, People's Republic of China; ^2^ Shandong Province Key Lab of Tumor Target Molecule, Jinan Central Hospital Affiliated to Shandong University, Jinan 250013, People's Republic of China

**Keywords:** targeting VEGFR drugs, safety, efficacy, meta-analysis, gastric cancer

## Abstract

The value of targeting VEGFR (vascular endothelial growth factor receptor) drugs has demonstrated encouraging anti-cancer activity in advanced solid tumors within current clinical trials. This study aimed to serve as the first systemic review to assess their safety and efficacy according to biochemical characteristics of targeting VEGFR drugs in gastric cancer. We analyzed eight clinical trials on targeting VEGFR drugs in gastric cancer. Results showed that targeting VEGFR drugs significantly improved overall survival (OS) [Hazard Ratio (HR) 0.69, 95% confidence interval (CI) (0.55, 0.83), *P <* 0.001], progression free survival (PFS) [HR 0.50, 95% CI (0.34, 0.66), *P <* 0.001], disease control rate (DCR) [Odds Ratio (OR) 3.83, 95% CI (2.39, 6.15), *P <* 0.001] and significantly decreased the progressive disease rate(PDR)[OR 0.45, 95% CI (0.34, 0.59), *P <* 0.001], but not objective response rate (ORR) [OR 1.46, 95% CI (0.93, 2.29), *P* = 0.098]. Further subgroup revealed that VEGFR antibody (VEGFR-Ab) drugs were superior to VEGFR tyrosine kinase inhibitor (VEGFR-TKI) drugs in terms of the OS, PFS and PDR. To determine the toxic effect of targeting VEGFR drugs, the relative risk of adverse events (grade ≥ 3) of special interest(AESIs) were estimated. Most of these were predictable and manageable. Furthermore, less AESIs were observed in the VEGFR-Ab than the VEGFR-TKI drugs. In conclusion, VEGFR drugs were effective targeted therapy in advanced or metastatic gastric cancer, and its toxicity is within a controllable range. VEGFR-Ab drugs were more effective than VEGFR-TKI drugs in terms of the OS, PFS and PDR of gastric cancer patients with little toxicity.

## INTRODUCTION

Gastric cancer is the third leading cause of cancer-related death worldwide. Nearly 1 million people worldwide are diagnosed with gastric cancer annually, and approximately half of them are in China [1]. First- and second-line chemotherapy has been demonstrated to provide survival benefit to patients with advanced or metastatic gastric cancer. Currently, the combination of fluorouracil and cisplatin has been identified as a standard first-line chemotherapy regimen for gastric cancer. Treatment with paclitaxcel weekly in combination with ramucirumab targeting vascular endothelial growth factor receptor 2 (VEGFR-2) is the first choice for second-line therapy [2]. Targeting VEGFR therapy has significantly improved long-term survival in advanced or metastatic gastric cancer.

Preclinical studies have confirmed that tumor metastasis or growth may be hindered in the absence of continuously promoting neovascularization [3, 4]. The vascular endothelial growth factor (VEGF) and its receptor (VEGFR) have been shown to play major roles in both physiological and tumor angiogenesis. The VEGF family consists of five ligands (VEGF-A, VEGF-B, VEGF-C, VEGF-D, and placental growth factor (PIGF)) and three receptor tyrosine kinases (VEGFR-1, -2, and -3). Of the VEGF receptors, VEGFR-2 expression is restricted to vasculature and appears to play a key role in angiogenesis [5, 6]. The VEGF-VEGFR system is an important target for anti-angiogenic therapy in cancer [7]. In molecular targeted therapy for gastric cancer, targeting VEGFR drugs have made a substantial breakthrough. For example, On April 21, 2014, the United States Food and Drug Administration (FDA) approved ramucirumab (Cyramza; Eli Lilly and Company) as monotherapy for the treatment of patients with advanced gastric or gastroesophageal junction adenocarcinoma with failure after prior treatment with first-line chemotherapy, and subsequently, on November 5, 2014, as combination therapy with paclitaxel [8]. Targeting VEGFR drugs mainly consist of VEGFR-Ab and VEGFR-TKI. However, no article has studied which is more safe and effective.

In this review, the targeting VEGFR drugs that meet the inclusion criteria are ramucirumab, regorafenib, apatinib, sunitinib, and TSU-68 (orantinib) [3, 9, 10]. Ramucirumab, a fully humanized immunoglobulin G-1 (IgG1) monoclonal antibody, prevents the binding of the VEGF ligand to the VEGFR-2 [6, 11]. Regorafenib, a multikinase inhibitor that targets various signaling pathways, such as VEGFR1/2/3, has been shown to efficiently inhibit tumor growth and angiogenesis in preclinical and clinical studies [12]. Sunitinib, an oral multi-target TKI with anti-VEGFR activity, blocks angiogenesis [13]. Apatinib, the latest orally administered TKI that selectively targets VEGFR-2 has encouraging preclinical and clinical data in the treatment of a variety of solid tumors. Apatinib was approved and launched in the People's Republic of China in 2014 as a subsequent-line treatment for patients with advanced gastric cancer [14]. TSU-68, a novel multiple tyrosine kinase inhibitor, inhibits VEGFR-2, platelet-derived growth factor receptor, and fibroblast growth factor receptor. Toi *et al.* showed that TSU-68, in combination with docetaxel, has a promising anti-tumor response with manageable toxicity in patients with anthracycline-resistant metastatic breast cancer [15]. Clinical trials have shown that targeting VEGFR drugs have a surprising anti-tumor activity in advanced solid tumors. For example, ramucirumab plus FOLFIRI (leucovorin, fluorouracil, and irinotecan) vs placebo plus FOLFIRI significantly improved overall survival [OS: HR 0.884, 95% CI (0.730, 0.976), *P* = 0.0219] compared with placebo plus FOLFIRI as second-line treatment for patients with metastatic colorectal carcinoma in a clinical trial known as RAISE [16].

The efficacy of targeting VEGFR drugs has also been demonstrated in gastric cancer. As mentioned above, ramucirumab was approved by the FDA in 2014 for the treatment of advanced gastric or gastroesophageal junction adenocarcinoma. However, some phase I/II studies of targeting VEGFR-TKI drugs do not show satisfactory outcomes when added to chemotherapy [17–20]. Meanwhile, in studies of single agents, there is evidence to suggest that the safety profiles of anti-angiogenic antibodies that targeting VEGFR-2 differ from those that VEGFR-TKI [21]. To date, there is no evidence-based systematic review on the safety and efficacy of targeting VEGFR drugs, including comprehensive comparison of VEGFR-Ab and VEGFR-TKI, in treating advanced or metastatic gastric cancer. It is urgent and important to summarize those results, offering evidence-based references for clinicians. This meta-analysis focused on the safety and efficacy of targeting VEGFR drugs in the treatment of advanced or metastatic gastric cancer based on prospective clinical trials.

## METHODS

### Search strategy

This systemic review and meta-analysis is reported in accordance with the Preferred Reporting Items for Systemic Reviews and Meta-Analyses (PRISMA) statement and was registered at the International Prospective Register of Systemic Reviews (number CRD 42017060812) [22].

All relevant studies were identified through the following computerized bibliographic databases: PubMed, Embase, Cochrane Library, ClinicalTrials.gov, EU Clinical Trials Register and Japan Pharmaceutical Information Center without any language restrictions (up to March 15, 2017). The following free language terms and medical subject headings (MeSH) were used as the specific search strategy: “Receptors, Vascular Endothelial Growth Factor”, “Stomach Neoplasms” and “Clinical Trial”. The complete search used for PubMed could be seen in at the end of the article appendix. All potentially eligible studies were temporarily considered for the review, regardless of its study design, language, or primary outcome. Additionally, we also performed clinical manual searches for references of relevant studies such as ASCO (America society of clinical oncology), in order to find additional publications in English.

### Study selection

The studies were identified according to the following inclusion criteria: (1) participants with advanced or metastatic gastric cancer or gastroesophageal junction cancer; (2) inhibitor of VEGFR as an experimental drug; (3) presence of the control group (placebo with or without chemotherapy) was used for comparison; (4) studies must report any of the following information: OS, PFS, ORR, DCR, PDR and AESIs. The exclusion criteria were: (1) insufficient data were available to estimate the outcomes; (2) observational and retrospective studies or animal studies; (3) the size of each arm was less than 10 participants; (4) no randomized studies; (5) not the study of targeting VEGFR drugs, such as VFGF antibody.

### Data extraction

Two independent investigators (Duanrui Liu, Yanfei Jia) reviewed study titles and abstracts. After the elimination of duplicates full texts were downloaded and assessed according to the following criteria for eligibility. Trials selected for detailed analysis and data extraction were analyzed by two investigators (Duanrui Liu, Yanfei Jia) with an agreement value of 95%. Disagreements were adjudicated by a third investigator (Yunshan Wang).

This meta-analysis exacted the following data from the studies that meet the inclusion criteria, including first author, publication year, number of patients, characteristics of patients, study design, intervention methods, ClinicalTrials.gov number, primary end point, line of trial, phase of trial, drug of biochemical characteristics, ORR, complete response rate (CRR), partial response rate (PRR), stable disease rate(SDR), DCR, PDR, median OS, median PFS, AESIs (grade ≥ 3) in each reported study. ORR was obtained directly from the study or calculated by CRR and PRR. Also, DCR was obtained directly from the study or calculated by CRR, PRR and SDR.

### Quality assessment

A systematic assessment of bias in the included trials was performed using the Cochrane criteria [23]. The entries used for the assessment of each study were as follows: random sequence generation, allocation concealment, blinding of participants and personnel, blinding of outcome assessment, incomplete outcome data, selective reporting and other bias. According to the recommendations of the Cochrane Handbook, a judgment to risk of bias was divided into three categories, including low risk, unclear risk and high risk.

### Statistical analysis

All data analysis was performed by STATA 12.0 or Revman 5.3. The safety was assessed by calculating overall risk of grade 3 to 4 AESIs (proteinuria, hypertension, bleeding or hemorrhage, and hand-foot syndrome symptoms). The efficacy of targeting VEGFR drugs in treating advanced or metastatic gastric cancer was assessed by calculating OS, PFS, ORR, DCR, PDR and HR or OR along with 95% CI based on data from all studies. Objective responses in included studies were measured according to the Response Evaluation Criteria in Solid Tumors (RECIST), version 1.0 or version 1.1, with modifications 15 and 16. In every included study, ORR% = [(complete responses + partial responses) ÷ total number of patients] × 100%, DCR% = [(complete responses + partial responses+ stable disease) ÷ total number of patients] × 100%. PDR is the percentage of patients whose cancer progress [24]. A 95% CI of HR, RR or OR not covering 1 or *P* value < 0.05 suggested the existence of statistical significance between the experiment group and the control group [25]. All indicators were presented with 95% CI. The heterogeneity among studies was assessed using the inconsistency index (*I*^2^) and chi-squared test. *P* values < 0.1 and *I*^2^ values > 50% suggested the existence of heterogeneity. If significant heterogeneity existed, we selected a random effect model; otherwise, the fixed-effects model was used. Meanwhile, *I*^2^ values of 25%, 50%, and 75% were considered to indicate low, moderate, and high heterogeneity, respectively [25].

### Sensitivity analysis and subgroup analysis

Sensitivity analysis was performed to search potential reasons that cause high heterogeneity. Moreover, we performed subgroup analysis to search the cause of heterogeneity according to the biochemical characteristics of targeting VEGFR drugs. And according to the level of heterogeneity, we choose random effect model or fixed effect model in order to yield a comparable pooled estimate.

### Publication bias

We assessed the possibility of publication bias by conducting Begg's and Egger's funnel plot asymmetry tests, and defined significant publication bias as a *p* value < 0.1 [26].

## RESULTS

### Study selection

**Figure 1:**
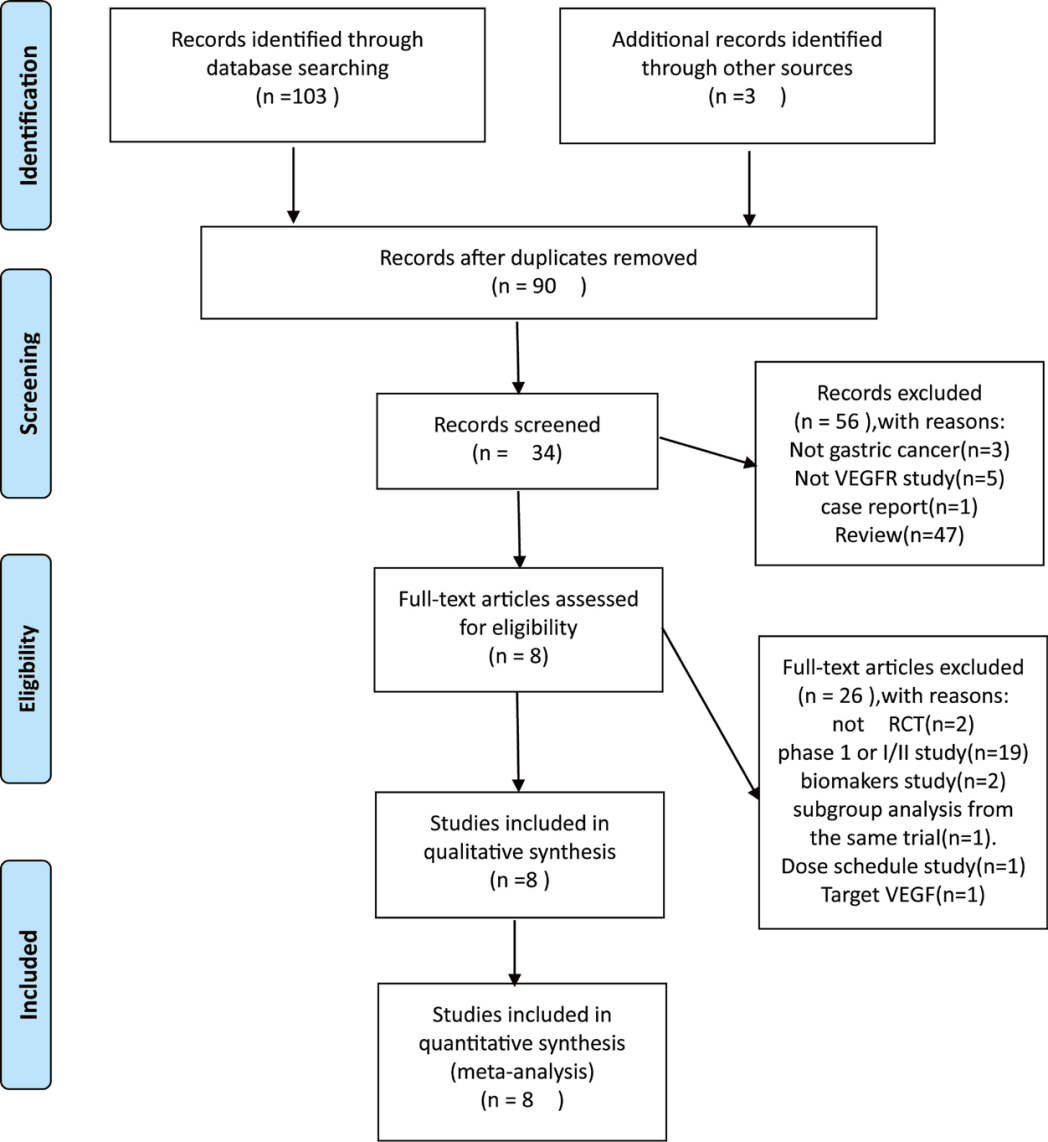
Study selection process

Our literature search yielded 106 potentially relevant articles. Sixteen studies were excluded as duplicates. By screening the title, abstract and keywords of each study according to inclusion and exclusion criteria, 56 studies were excluded. The full texts of 34 articles were then reviewed in detail. Eight studies were included in our meta-analysis by removing retrospective articles and reviews that were incompatible to inclusion criteria and those lacking necessary data. Among these studies, 3 conference abstracts were obtained by manually searching the American Society of Clinical ASCO. Finally, 8 studies [27–34] were included in our work. Study selection process was shown in Figure 1.

### Study characteristics

The characteristics of the studies included are summarized in Table 1. The included studies were published between 2013 and 2016. The 8 studies included 1,146 participants treated with targeting VEGFR drugs and 879 participants in the control arm for a total of 2,025 participants. Among these studies, 3 studies [29–31] researched an VEGFR-Ab drug (ramucirumab) as the experimental drug, and 5 studies [27, 28, 32–34] researched VEGFR-TKI drugs (apatinib, regorafenib, sunitinib, TSU-68) as the experimental drugs. The control arm consisted of placebo, chemotherapy, such as S-1/CDDP, and placebo plus chemotherapy, such as placebo+ FOLFIR. One study [27] researched two schedules of apatinib. There were also 4 phase II studies [27, 30, 32, 34], 3 phase III studies [29, 31, 33]. and 1 phase I/III study [28] in the included trials. Two trials [30, 32] were conducted in the first-line setting and the other six trials in the second through fourth line settings. There was only one clinical trial [32] registered at the Japan Pharmaceutical Information Center, and the remaining included clinical trials [27–31, 33, 34] in ClinicalTrial.gov.

**Table 1:** Characteristics of included trials

Study	Phase	Treatment arms	No. of patients (exp. arm /con. arm)	Line	No. of clinical trials.gov	Primary end point	Drug of biochemical characteristics	Characteristics of patients			
								Median Age(range)	Sex(male%)	race	ECOG PS distribution
REGARD(2014)[29]	III	Ramucirumab vs Placebo	238/117	2^nd^	NCT00917384	OS	VEGFR-Ab	60(52—67)^*^/ 60(51—57)^#^	71^*^/68^#^	16^*^/15^#^	72^*^/74^#^
Yoon(2016)[30]	II	Ramucirumab+mFOLFOX6 vs Placebo+mFOLFOX6	84/84	1^st^	NCT01246960	PFS	VEGFR-Ab	64.5(27—83) / 60(34—82)	75/72.6	2.4/4.8	51.2/48.8
RAINBOW (2014)[31]	III	Ramucirumab + paclitaxel vs Placebo +paclitaxel	330/335	2^nd^	NCT01170663	OS	VEGFR-Ab	61(25—83) / 61(24—84)	69/73	33/36	65/57
Yamaguchi et al (2013)[32]	II	s-1/CDDP+TSU-68 vs S-1/CDDP	47/45	1^st^	JapicCTI-101327	PFS	VEGFR-TKI	62(30—74) / 63.5(44—76)	66.7/76.1	All	37.8/34.8
Li J et al(2016)[28]	II/III	Apatiniba vs placebo	176/91	3^rd^/ 4^th^	NCT01512745	OS/PFS	VEGFR-TKI	58(23—71) / 58(28—70)	75/75.8	All	72.7/83.5
GRID(2016)[33]	III	Regorafenib vs placebo	133/66	3^rd^	NCT01271712	PFS	VEGFR-TKI	60(18—82) / 61(28—87)	63.9/63.6	25.6/24.2	45.1/43.9
Li J et al (2013)[27]	II	Apatinib vs placebo	47^+^ 46^++^/48	3^rd^ /4^th^	NCT00970138	PFS	VEGFR-TKI	54^a^/55^b^/53^c^ (NA)	75^a^/83^b^/74^c^	All	98^a^/94^b^/96^c^
Moehler et al (2016)[34]	II	Sunitinib+FOLFIRI vs placebo+FOLFIRI	45/45	2^nd^ /3^rd^	NCT01020630	PFS	VEGFR-TKI	62(37—76) / 57(28—84)	73/67	NA	NA

^+^:apatinib 850mg once a day; ^++^:apatinib 425mg twice a day. ^*^:exprimental arm ^#^:control arm.

Exp. arm: experimental arm; Con. arm: control arm; OS: overall survival; PFS: progression-free survival. ECOG PS: Eastern Cooperative Oncology Group performance status. VEGFR-Ab: vascular endothelial growth factor receptor antibody; VEGFR-TKI: vascular endothelial growth factor receptor tyrosine kinase inhibitors.

TS-1: tegafur, gimeracil, oteracil potassium, CDDP:cisplatin. All: all Asian. ^a^: 850mg once a day; ^b^:425mg twice a day; ^c^:placebo NA: not available.

### Assessment of methodological quality

**Figure 2:**
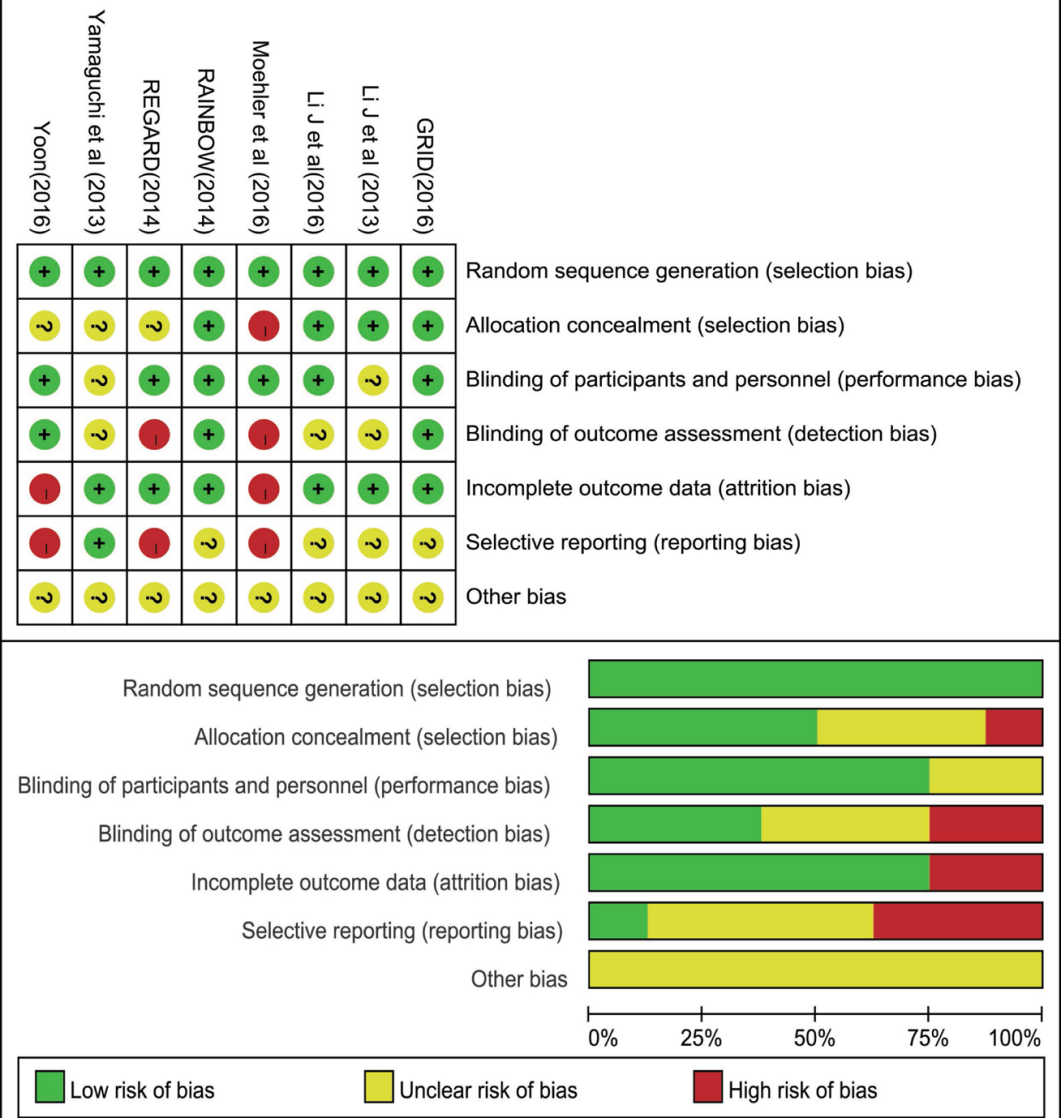
Accessment of risk of bias

We critically assessed the methodological quality of included studies in accordance with the Cochrane Collaboration risk of bias tool. All 8 trials reported adequate randomization, none was stopped early, and all of them were multicenter studies. Therefore, the 8 studies were rated as low bias risk in randomization. Other bias sources were not identified. The graphical results of methodological quality are shown in Figure 2.

### Efficacy analysis

Five indicators (OS, PFS, ORR, DCR and PDR) were used to measure the efficacy of targeting VEGFR drugs in treating advanced or metastatic gastric cancer. Characteristics of patients in analyzed trials are summarized in Table 1, and the main data of efficacy testing is shown in Table 2. Five of the 8 studies showed a significant improvement in OS, and 6 showed a significant improvement in PFS. Median OS in the experimental arm in included trials ranged from 4.3 to 16.6 months, and median PFS ranged from 2.1 to 6.9 months. Yamaguchi *et al.* (2013) [32] reported the highest ORR (62.2%) of the 8 eight trials in experimental group, and *Yoon* (2016) [30] reported the best DCR (84.5%) in all the studies. Ramucircumab was the research drug used in the above two trials [22, 24]. The pooled results with random effect analysis revealed that compared to the control arm, treatment with targeting VEGFR drugs improved PFS. [HR 0.50, 95% CI (0.34, 0.66) (*P <* 0.001, Figure 4A) and prolonged OS [HR 0.69, 95% CI (0.55, 0.83) (*P <* 0.001, Figure 4B), respectively. However, analysis of all included trials showed that treatment with addition of targeting VEGFR drugs had an OR of 1.46 (95% CI: 0.93–2.29, *P* = 0.098, Figure 4C) not significantly improved ORR compared to the control arm. And the pooled response rate was 17% (195/1144) in the experimental arm and 15.5% (136/880) in the control arm. DCR was improved with an OR of 4.29 [95% CI (2.47, 7.46), *P <* 0.001, Figure 4D]. The pooled DCR was 60.2% (662/1099) in the experimental arm and 41.4% (345/834) in the control arm. Meanwhile, targeting VEGFR drugs had a significant trend of decreasing PDR compared with the control arms (OR 0.45, 95% CI (0.34, 0.59), *P <* 0.001, Figure 5).

**Table 2:** Four efficacy indicators of TVDs in the included studies

study	TVDs	OS				PFS				ORR (%)	DCR (%)
		mOS (months)	HR	95%CI	*P*	mPFS (months)	HR	95%CI	*P*		
REGARD (2014) [29]	Ram	5.2^a^/3.8^b^	0.78	0.603—0.998	0.047	2.1^a^/1.3^b^	0.48	0.376—0.420	<0.001	3^a^/3^b^	49^a^/23^b^
Yoon (2016) [30]	Ram	11.7/11.5	10.80	0.730—1.580	0.712	6.4/6.7	0.98	0.690—1.370	0.886	45.2/46.6	84.5/66.7
RAINBOW (2014) [31]	Ram	9.6/7.4	0.81	0.678—0.962	0.017	4.4/2.9	0.64	0.536—0.752	<0.001	27.9/16.1	80/63.6
Yamaguchi *et al.* (2013) [32]	TSU-68	16.6/15.5	0.74	0.460—1.190	0.213	6.9/7.1	1.23	0.740—2.050	0.424	62.2/56.5	NA
Li J *et al.* (2016) [28]	Apa	6.5/4.7	0.71	0.537—0.937	0.016	2.6/1.8	0.44	0.331—0.595	<0.001	2.8/0	42.1/8.8
GRID (2016) [33]	Reg	NA	0.77	0.423—1.408	0.199	4.8/0.9	0.27	0.185—0.388	<0.001	4.5/1.5	52.6/9.1
Li J *et al.* (2013) [27]	Apa (850mg qd)	4.9/2.5	0.37	0.220–0.620	<0.001	3.7/1.4	0.18	0.100—0.340	<0.001	6.4/0	51.1/10.4
	Apa (425mg bid)	4.3/2.5	0.41	0.240—0.720	0.002	3.2/1.4	0.21	0.110—0.380	<0.001	13.0/0	35.8/10.4
Moehler *et al.* (2016) [34]	Sun	10.4/8.9	0.82	0.500–1.340	0.420	3.6/3.3	1.11	0.700—1.740	0.660	20.0/29.0	60.0/56.0

^a^: experimental arm; ^b^: control arm.

OS: overall survival; PFS: progression-free survival; mOS: median overall survival; HR: hazard ratio; CI: confidence interval; ORR: overall objective rate; DCR: disease control rate; TVDs: targeting VEGFR drugs; NA: not available; Ram: ramucirumab; Apa: apatinib; Sun: sunitib.

**Figure 3:**
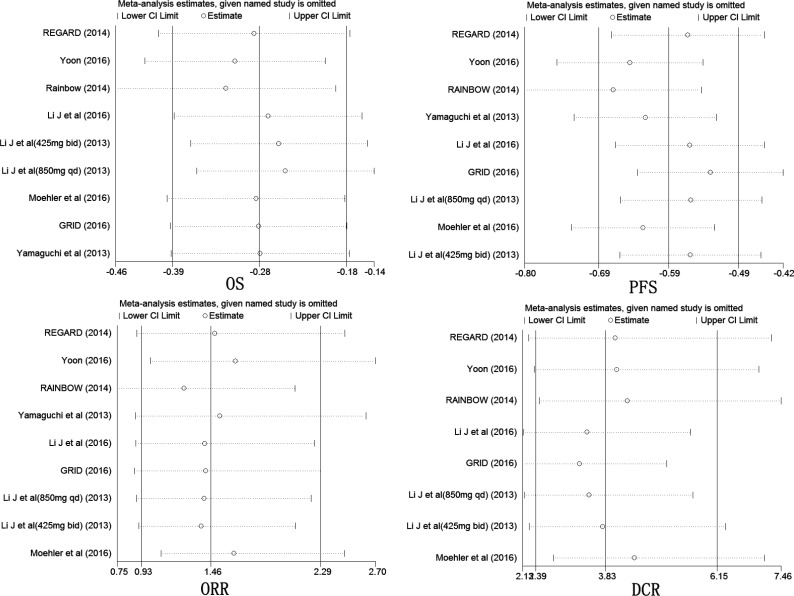
Sensitivity analysis of the summary HR of PFS, OS, ORR and DCR The results were computed by omitting each study in turn. Random effects estimates (exponential form) were used in analysis. The two ends of the dotted lines represent the 95% CI.

**Figure 4:**
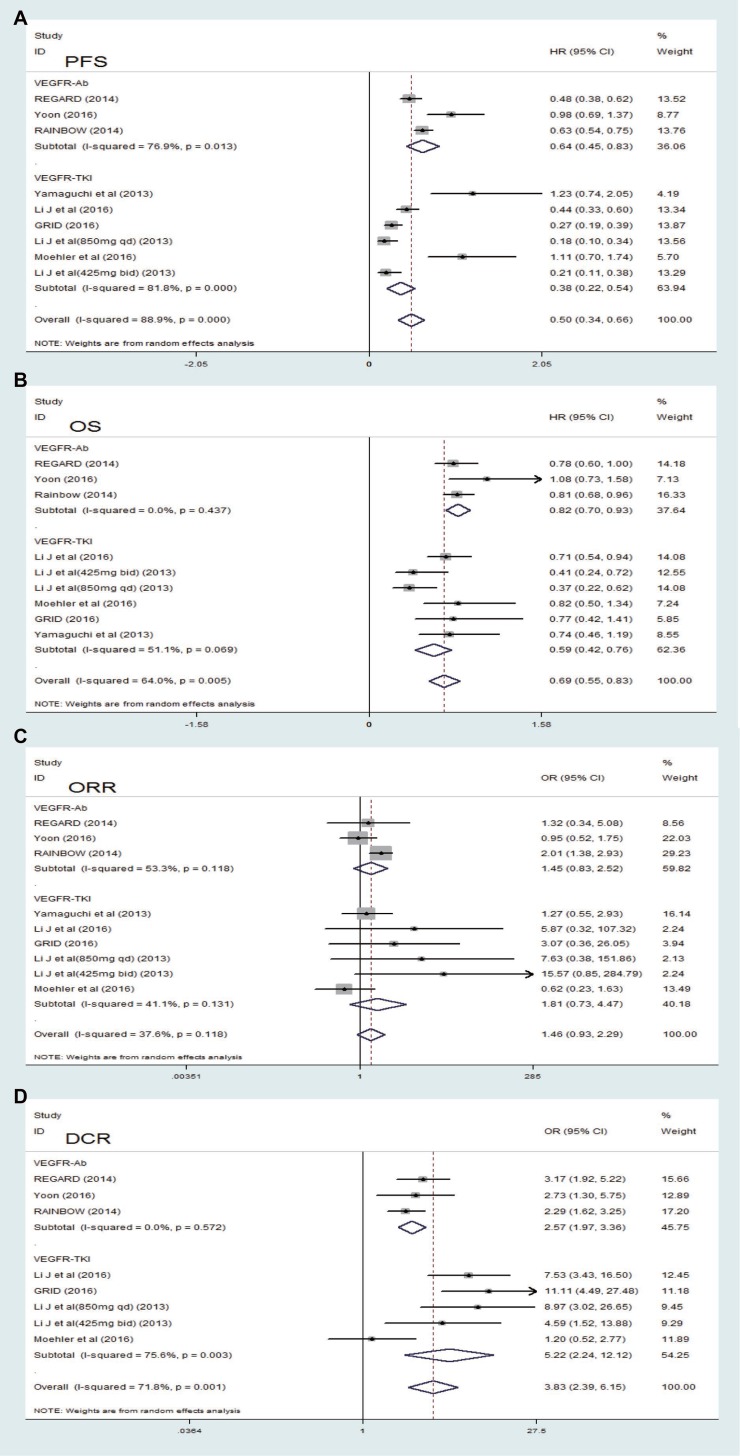
Forest plot and pooled HR and 95 % CI for PFS (A), OS (B), ORR (C) and DCR (D) in overall and subgroups The pooled HR for overall and subgroups showed that the patients receiving targeting VEGFR drugs therapy possessed a significant improvement in PFS and OS. The pooled OR for ORR and DCR in overall and subgroups showed that the patients receiving VEGFR drug therapy possessed a significant improvement. HR hazard ratios, OR odds ratio, OS overall survival, PFS progression-free survival, CI confidence intervals, VEGFR-TKI VEGF-Receptor Tyrosine kinase inhibitor, VEGFR-Ab VEGF-Receptor antibody.

**Figure 5:**
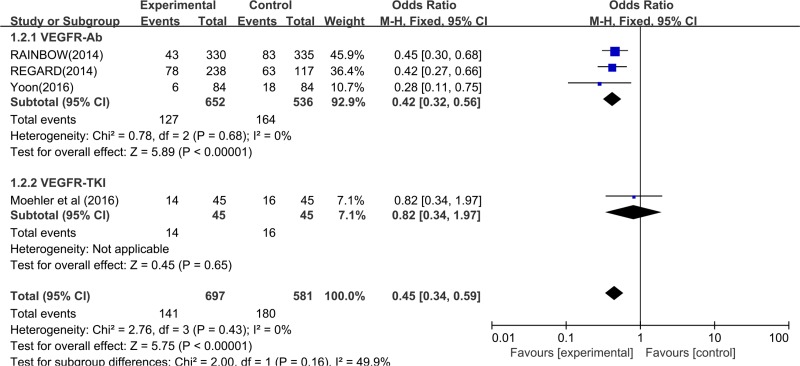
Forest plot and pooled odds ratio (OR) and 95 % CI for PDR in overall and subgroups PDR progressive disease rate, CI confidence intervals, VEGFR-TKI VEGF-Receptor Tyrosine kinase inhibitor, VEGFR-Ab VEGF-Receptor antibody.

### Safety analysis

The toxicity reported in included studies was shown in Table 3 (only grade ≥3 AESIs was present). Overall, in addition to common toxicity of chemotherapy, the incidence of special toxicity associates with targeting VEGFR drugs could reflect its safety, including hypertension, bleeding or hemorrhage, arterial thromboembolic events (ATE), venous thromboembolic events (VTE), proteinuria, hand-foot syndrome, gastrointestinal (GI) perforation, renal failure, cardiac failure, and infusion-related reaction. In all studied AESIs that co-reported in two subgroups, the incidence of hypertension [RR 5.54, 95% CI (3.38, 9.07), *P <* 0.001] was the highest. We also found that hypertension, proteinuria [RR 4.50, 95% CI (1.20, 16.83), *P* = 0.026] and hand-foot syndrome [RR 16.21, 95% CI (3.77, 69.67), *P <* 0.001] were significantly increased in patients treated with targeting VEGFR drugs. And there were no statistically significant differences in bleeding or hemorrhage, ATE, VTE, GI perforation, renal failure, cardiac failure, and infusion-related reaction.

**Table 3:** Subgroup analysis and RR of AESIs (grade ≥ 3) in the included studies

AESIs (grade ≥3) of special interest	No. of total studies	Events/total in VEGFR-Ab subgroup		Events/total in VEGFR-TKI subgroup		Total group		
		Exp. arm	Con. arm	Exp. arm	Con. arm	RR(95% CI)	*P* value	Effect model
Hypertension	6	79/645	15/524	48/402	2/205	5.54 (3.38,9.07)	<0.001	Fixed
Bleeding or haemorrhage	4	27/645	16/524	6/176	7/91	1.07 (0.64,1.80)	0.778	Fixed
ATE	3	8/645	3/524	NR	NR	1.87 (0.56,6.28)	0.313	Fixed
VTE	3	14/645	20/524	NR	NR	0.57 (0.31,1.15)	0.125	Fixed
proteriuria	4	5/563	0/444	7/269	0/139	4.50 (1.20,16.83)	0.026	Fixed
hand-foot syndrome	3	NR	NR	49/402	0/205	16.21 (3.77,69.67)	<0.001	Fixed
GI perforation	2	6/563	1/444	NR	NR	3.16 (0.61,16.45)	0.172	Fixed
Renal failure	2	6/409	4/409	NR	NR	1.44 (0.44,4.76)	0.546	Fixed
Cardiac failure	2	2/563	2/444	NR	NR	1.01 (0.14,7.10)	0.995	Fixed
Infusion-related reaction	2	2/563	0/444	NR	NR	5.03 (0.24,104.38)	0.296	Fixed

Exp.arm: experimental arm; Con. arm: control arm; RR: relative risk; ATE: arterial thromboembolic events; VTE: venous thromboembolic events; GI: gastrointestinal; Fixed: Fixed effect model; NR: not report.

### Sensitivity analysis

Significant heterogeneity was detected among the studies in OS (*I*^2^ = 64%, *P* = 0.005, Figure 4B), PFS (*I*^2^ = 88.9%, *P <* 0.001, Figure 4A), ORR (*I*^2^ = 37.6%. *P* = 0.118, Figure 4C) and DCR (*I*^2^ = 71.8%, *p* = 0.001, Figure 4D), except PDR (*I*^2^ = 0%, *P* = 0.48, Figure 5). Therefore, we conducted a sensitivity analysis. As shown in Figure 3, no article was found to be beyond the limits in OS, PFS, ORR and DCR, far from the scope of other studies, which would have helped to identify heterogeneity. Sensitivity analysis suggested that our results are stable.

### Subgroup analysis

We speculated that the biochemical characteristics of targeting VEGFR drugs included in the clinical trials led to high heterogeneity. All the included studies were divided into two subgroups, VEGFR-Ab and VEGFR-TKI, according to biochemical characteristics, respectively. As shown in Figure 4B for the analysis of OS, the pooled HR was 0.82 [95% CI (0.70, 0.93), *P <* 0.001] with low heterogeneity (*I*^2^ < 0.01, *P* = 0.437) in the VEGFR-Ab subgroup, and the pooled HR was 0.59 [95% CI (0.42, 0.76), *P <* 0.001] with mediate heterogeneity (*I*^2^ = 51.1%, *P* = 0.069) in the VEGFR-TKI subgroup, respectively. We can also conclude that VEGFR-Ab subgroup have a better OS benefit than the VEGFR-TKI subgroup. For the analysis of PFS (Figure 4A), the pooled HR was 0.64 [95% CI (0.45, 0.83), *P <* 0.001], with a high heterogeneity (*I*^2^ = 76.9%, *P* = 0.013) in the VEGFR-Ab subgroup and the pooled HR was 0.38 [95% CI (0.22, 0.54), *P <* 0.001] with a high heterogeneity (*I*^2^ = 81.8%, *P <* 0.001) in the VEGFR-TKI subgroup, respectively. Compare to the VEGFR-TKI subgroup, a greater PFS benefit was found in the VEGFR-Ab subgroup. In the subgroup analysis of DCR, the VEGFR-TKI subgroup [OR 5.22, 95% CI (2.24, 12.12), *P <* 0.001, Figure 4D] exhibited a DCR higher significantly than that of the VEGFR-Ab subgroup [OR 2.57, 95% CI (1.97, 3.36), *P <* 0.001, Figure 4D]. There was a low heterogeneity in the VEGFR-Ab subgroup, but not in the VEGFR-TKI subgroup (Figure 4D). However, in subgroup analysis of ORR, both the VEGFR-Ab subgroup [OR 1.45, 95% CI (0.83, 2.52), *P* = 0.195, Figure 4C] and the VEGFR-TKI subgroup [OR1.81, 95% CI (0.73, 4.47), *P* = 0.198, Figure 4C] showed not significantly improved ORR. There was a high heterogeneity in the VEGFR-Ab subgroup and a mediate heterogeneity in the VEGFR-TKI subgroup (Figure 4C). Therefore, a random effect model was used for all above analysis in order to yield a comparable pooled estimate. Data for PDR were available from four trials [29–31, 34]. The pooled OR for PFS demonstrated that VEGFR-Ab was associated with significantly lower PDR when compared with VEGFR-TKI in treatment for the patients with advanced or metastatic gastric cancer [VEGFR-Ab: OR 0.42, 95% CI (0.32, 0.56), *P <* 0.0001, Figure 5]. A fixed effect model was used because low heterogeneity was found between the trials (VEGFR-Ab: I^2^=0%, *P* = 0.68).

In subgroup analysis of AESIs (Table 3), the RR of hand-foot syndrome was highest and only reported in the VEGFR-TKI subgroup. However, ATE [RR 1.87, 95% CI (0.56, 6.28), *P* = 0.313], VTE [RR 0.57, 95% CI (0.31, 1.15), *P* = 0.125], GI perforation [RR 3.16, 95% CI (0.61, 16.45), *P* = 0.172], renal failure [RR 1.44, 95% CI (0.44, 4.76), *P* = 0.546], cardiac failure [RR 1.01, 95% CI (0.14, 7.10), *P* = 0.995], and infusion-related reaction [RR 5.03, 95% CI (0.24, 104.38), *P* = 0.296] were not reported in the VEGFR-TKI subgroup. The pooled analysis (AESIs occurred ≥ 2 trials) showed that the risk of hypertension [RR 4.68, 95% CI (2.68, 8.17), *P <* 0.001, *I*^2^ *<* 0.1] in the VEGFR-Ab subgroup was lower than that of the VEGFR-TKI subgroup [RR 8.49, 95% CI (2.92, 24.73), *P <* 0.001, *I*^2^ *<* 0.1], and the risk of proteinuria [RR 3.96, 95% CI (0.46, 34.10), *P* = 0.211, I^2^ *<* 0.1] in both the VEGFR-Ab and VEGFR-TKI subgroups [RR 4.27, 95% CI (0.74, 24.49), *P* = 0.104, I^2^ *<* 0.1]was not significantly higher than that of the control arm.

### Publication bias

There was no evidence of significant publication bias in OS, PFS, ORR, DCR and PDR, as shown in the formal statistical tests: (1) PFS: Egger's test, *P* = 0.544; Begg's test, *P* = 0.602; (2) OS: Egger's test, *P* = 0.251; Begg's test, *P* = 0.251; (3) ORR: Egger's test, *P* = 0.560; Begg's test, *P* = 0.175. (4) DCR: Egger's test, *P* = 0.142; Begg's test, *P* = 0.386. (5) PDR: Egger's test, *P* = 0.881; Begg's test, *P* = 0.734.

## DISCUSSION

This is the first systematic review and meta-analysis to comprehensively evaluate the efficacy and safety of targeting VEGFR drugs, including VEGFR-Ab and VEGFR-TKI drugs, for advanced or metastatic gastric cancer. The present results demonstrated that treatment with targeting VEGFR drugs significantly improved OS, PFS, DCR and significantly decreased PDR in advanced or metastatic gastric cancer, except ORR. The odds of drug-related toxicity (grade ≥ 3) were also significantly increased, including hypertension, proteinuria and hand-foot syndrome. In the subgroup analysis, according to the biochemical characteristics of drug, the VEGFR-Ab subgroup achieved a higher OS [HR 0.82, 95% CI (0.70, 0.93), *P <* 0.001] and PFS [HR 0.59, 95%CI (0.42, 0.76), *P <* 0.001] than the VEGFR-TKI subgroup, but opposite results for DCR (VEGFR-TKI: OR 5.22, 95% CI (2.24, 12.12), *P <* 0.001]. Meanwhile, the pooled OR of PDR indicated that the VEGFR-Ab subgroup was superior to the VEGFR-TKI subgroup. However, both the VEGFR-Ab subgroup [OR 1.45, 95% CI (0.83, 2.52), *P* = 0.195] and the VEGFR-TKI subgroup [OR1.81, 95% CI (0.73, 4.47), *P* = 0.198] showed not significantly improved ORR. In terms of toxicity, the use of VEGFR-TKI drugs has shown more AESIs (grade ≥3) than that the VEGFR-Ab subgroup in addition to common toxicity of chemotherapy, and a high incidence of hand-foot syndrome was only shown in the VEGFR-TKI subgroup. From the AESIs (occurred ≥ 2 trials in both subgroups), the risk of hypertension was higher in the VEGFR-TKI subgroup than in the VEGFR-Ab subgroup, and the risk of proteinuria was not found to be significantly improved in the experimental arm than in the control arm. Therefore, we concluded that the safety of VEGFR-Ab drugs was better than VEGFR-TKI drugs overall.

In the management of gastric cancer, chemotherapy is currently the main treatment. However, there is no standard first-line chemotherapy regimen to choose. Furthermore, traditional chemotherapy did not achieve long-term stable effects. Therefore, it is necessary to explore new treatment programs for advanced or metastatic gastric cancer. In the last 10 years, the molecular targeted therapy for gastric cancer has achieved remarkable success. Among the novel targeted therapy strategies, study of anti-angiogenesis is more extensive and in-depth. A series of studies have shown that the angiogenesis pathway modulated by the VEGF family in many tumors contributes to the progression, invasion, and metastasis of malignancy and inhibits malignant tumor growth [35]. Thus, targeting the VEGF/VEGFR pathway receives more attention as result of the survival outcome superior to traditional chemotherapy from phase III clinical trials [29, 31, 33]. Some research and clinical trials [27–29, 31, 36–38] have showed anti-VEGFR inhibitor treatment was more efficacious than anti-VEGF treatment in terms of OS and PFS. In our study, we focus on analyzing the safety and efficacy of the VEGFR inhibitors from the characteristics of each drug in gastric cancer.

There are five targeting VEGFR drugs included in our meta-analysis, consisting of ramucirumab, apatinib, regorafenib, and sunitinib, TSU-68 (orantinib). Ramucirumab, a monoclonal antibody, can selectively combine with VEGFR-2 and inhibit the downstream effects of the VEGF pathway in angiogenesis. The REGARD [29] and RAINBOW [31] trials reported the superior benefits of ramucirumab [OS_REGARD_: HR 0.78, 95% CI (0.603, 0.998), *P* = 0.047, OS_RAINBOW_: HR 0.81, 95% CI (0.678, 0.962), P =0.017, respectively; and PFS_REGARD_: HR 0.48, 95% CI (0.376, 0.620), *P <* 0.001, PFS_RAINBOW_: HR 0.64, 95% CI (0.536, 0.752), *P <* 0.001, respectively]. Based on the superior efficacy of ramucirumab, it was approved by the FDA to treat patients with advanced gastric or gastroesophageal junction adenocarcinoma with failure after prior treatment with first-line chemotherapy on 2014. VEGFR-TKIs, including apatinib, regorafenib, sunitinib, and TSU-68 (orantinib), attracted much attention due to their efficacy and tolerable toxicity. For example, apatinib, a small-molecule VEGFR tyrosine kinase inhibitor, reported an OS [HR0.71, 95% CI (0.537, 0.937), *P* = 0.016] and PFS [HR 0.44, 95% CI (0.331, 0.595), *P <* 0.001] advantage in favor of the experimental treatment in a trial by Li *et al.* [28]. Our study has also demonstrated that treatment with targeting VEGFR drugs significantly improves outcomes in terms of OS, PFS, DCR and PDR in patients with advanced or metastatic gastric cancer.

In addition to efficacy, safety and tolerability are our anther focus in the treatment of gastric cancer. The most frequent grade ≥ 3 TESIs are summarized in Table 3. Most TESIs or TEAEs (treatment emergent adverse events) are predictable and within a controllable range. However, hypertension, proteinuria and hand-foot syndrome were significantly increased in patients treated with targeting VEGFR drugs. Due to inhibition of targets in part that do not necessarily involved VEGF/VEGFR signaling, VEGFR inhibitors frequently induce toxicities such as hypertension, fatigue, delayed wound healing [3, 39]. Treatment-related hypertension is dose dependent and reflects on-target inhibition rather than off-target effects, which closely correlated with the potency of VEGFR-2 inhibition [40]. In AESIs, we also speculate that the risk of proteinuria is significant increase in the treatment with targeting VEGFR drugs, which also associated with the effects of targeting VEGFR drugs on endothelial cells. To our regret, there have been no confirmatory studies to discover robust predictable biomarker to improve therapeutic approaches. However, some clinical trials bring us some hope. For example, in multiple phase II studies of sunitinib, high sKIT and low VEGF-C were significantly associated with clinical benefit [18].

As VEGFR-Ab and VEGFR-TKI drugs showed different biochemical characteristics and the different profile of safety, we conducted subgroup analysis, which may guide clinical decision-making in the use of a specific agent in an individual patient. VEGFR-Ab drugs only bind to VEGFR while VEGFR–TKIs target a wide number of tyrosine receptor and kinases. In the subgroup analysis, the VEGFR-Ab subgroup achieved a higher OS and PFS than the VEGFR-TKI subgroup. Meanwhile, the pooled OR of PDR indicated that the VEGFR-Ab subgroup was superior to the VEGFR-TKI subgroup. Furthermore, the safety profiles of VEGF antibodies differ from TKI. Compared with VEGFR-Ab, VEGFR-TKI is generally related with a higher incidence of hematologic TEAEs, especially leukopenia, neutropenia, thrombocytopenia, elevated aminotransferase and anemia [27, 28, 32–34]. The hand-foot syndrome is a painful erythema, often preceded by paresthesia that is a toxic reaction often related to some cytotoxic agents like doxorubicin, docetaxel, and fluorouracil/capecitabine [41]. Because VEGF has physiological roles to play in mucosal integrity and neuronal functioning, it is believable that inhibiting VEGF or VEGFR could induce a combined deficit that translates into this side-effect [42]. In this meta-analysis, the hand-foot syndrome is only reported in VEGFR-TKI subgroup.

This study represents an important step forward in gastric cancer study, because it proved the value of targeting VEGFR drugs, including VEGFR-Ab and VEGFR-TKI, on the basis of available evidence that supports the use of ramucirumab [43]. More recently, a mate-analysis of five trials on targeting VEGFR-2 in a total of 1,596 patients with advanced gastric cancer demonstrated that anti-VEGFR-2 inhibitors revealed a significant increase in OS [HR: 0.69, 95% CI (0.55, 0.87) *p* = 0.002] [44]. However, this analysis, which included only a small number of trials and only studied VEGFR-2, cannot reflect the overall efficacy of targeting VEGFR drugs and did not have a subgroup analysis to detect the cause of high heterogeneity. Yu *et al.* [36] analyzed antiangiogenic treatment in patients with advanced GC before 2016. Meanwhile, they paid little attention to the biochemical characteristics of drugs and lacked systematic analysis of VEGFR drugs. With the completion of new clinical trials for targeting VEGFR drugs, we analyzed the safety and efficacy of the whole and subgroups from the characteristics of each drug.

There are a few limitations to our study: (1) the most significant limitation is the reliance on data in the public domain that leads to the risk of publication bias. However, the results of Egger's and Begg's tests revealed a low likelihood of publication bias. (2) The analysis was conducted in a large sample size (only eight trials) and based on the present literature rather than on the data of individual patients. This might have introduced some biases to the final consequence. (3) Only a small number of trials were available. (4) The adverse events (AEs) were limited to grade ≥ 3 AESIs.

## CONCLUSIONS

In conclusion, our review has identified that treatment with targeting VEGFR drugs significantly improves outcomes in terms of OS, PFS, DCR and PDR in patients with advanced or metastatic gastric cancer, and its toxicity is within a controllable range. VEGFR-Ab drugs were more effective than VEGFR-TKI drugs in terms of the OS, PFS and PDR of gastric cancer patients with little toxicity, but they are weak in increasing the DCR. Further research is necessary to confirm these findings and detect the potentially predictive biomarkers of targeting VEGFR drugs to choose the best treatment and improve clinical benefit.

## Appendix

The complete search used for PubMed was: (“Receptors, Vascular Endothelial Growth Factor” [MeSH Terms] OR “Receptors, Vascular Endothelial Growth Factor” [Free Words] OR “Receptor, Vascular Endothelial Cell Growth Factor Receptor” OR “Vascular Permeability Factor” [Free Words] OR “VEGF Receptors” [Free Words] OR “Receptors, VEGF” [Free Words] OR “Vascular Endothelial Cell Growth Factor Receptor” [Free Words] OR “VPF Receptor” [Free Words] OR “Vascular Endothelial Growth Factor Receptor” [Free Words] OR “VEGF Receptor” [Free Words] OR “Vascular Permeability Factor Receptor” [Free Words] OR “Endothelial Growth Factor Receptor” [Free Words] OR “Receptor, Endothelial Growth Factors” [Free Words]) AND (“Stomach Neoplasms” [MeSH Terms] OR “Neoplasm, Stomach” [Free Words] OR “Neoplasms, Stomach” [Free Words] OR “Gastric Neoplasms” [Free Words] OR “Gastric Neoplasm” [Free Words] OR “Neoplasm, Gastric” [Free Words] OR “Neoplasms, Gastric” [Free Words] OR “Cancer of Stomach” [Free Words] OR “Stomach Cancers” [Free Words] OR “Gastric Cancer” [Free Words] OR “Cancer, Gastric” [Free Words] OR “Cancers, Gastric” [Free Words] OR “Gastric Cancers” [Free Words] OR “Stomach Cancer” [Free Words] OR “Cancer, Stomach” [Free Words] OR “Cancers, Stomach” [Free Words] OR “Cancer of the Stomach” [Free Words] OR “Gastric Cancer, Familial Diffuse” [Free Words]) AND (“Clinical Trial” [MeSH Terms] OR “Clinical Trial” [Free Words] OR “Clinical Study” [Free Words] OR “Clinical Trial, Phase I” OR “Clinical Trial, Phase II” [Free Words] OR “Clinical Trial, Phase III” [Free Words] OR “Clinical Trial, Phase IV” [Free Words] OR “Controlled Clinical Trial” [Free Words] OR “Randomized Controlled Trial” [Free Words]).
